# Transfer Learning Based Method for Frequency Response Model Updating with Insufficient Data

**DOI:** 10.3390/s20195615

**Published:** 2020-10-01

**Authors:** Zhongmin Deng, Xinjie Zhang, Yanlin Zhao

**Affiliations:** School of Astronautics, Beihang University, Beijing 100191, China; dengzhongmin@buaa.edu.cn (Z.D.); zhaoyanlin@buaa.edu.cn (Y.Z.)

**Keywords:** model updating, frequency response, deep convolutional neural network, transfer learning, domain adaptation

## Abstract

Finite element model updating precision depends heavily on sufficient vibration feature extraction. However, adequate amount of sample collection is generally time-consuming in frequency response (FR) model updating. Accurate vibration feature extraction with insufficient data has become a significant challenge in FR model updating. To update the finite element model with a small dataset, a novel approach based on transfer learning is firstly proposed in this paper. A readily available fault diagnosis dataset is selected as ancillary knowledge to train a high-precision mapping from FR data to updating parameters. The proposed transfer learning network is constructed with two branches: source and target domain feature extractor. Considering about the cross-domain feature discrepancy, a domain adaptation method is designed by embedding the extracted features into a shared feature space to train a reliable model updating framework. The proposed method is verified by a simulated satellite example. The comparison results manifest that sample amount dependency has prominently lessened this method and the updated model outperforms the method without transfer learning in accuracy with the small dataset. Furthermore, the updated model is validated through dynamic response out of the training set.

## 1. Introduction

Model updating is an important topic in dynamic analysis and structural engineering [1,2], which is aimed at improving the finite element model reliability. Frequency response (FR) is commonly regarded as the updating objective in the model updating algorithm [3,4,5]. The model updating performance overwhelmingly relies on the sample amount [6,7]. Especially in methods based on the deep neural network [8], the requirement of the training sample amount is generally extensive. Unfortunately, adequate sample collection is extremely time-consuming in practice [9,10]. Insufficient data problems become a common obstacle in FR model updating [11] and reducing sample dependency would be desirable.

However, in previous model updating methods, researchers generally replace the repeated FR data calculation by some approximate functions to address the small sample problem [12,13]. The complicated dynamic propagation function is roughly displaced by a simplified formula. Fang et al., 2015, employed the polynomial-based response surface model to reduce repeated natural frequency calculation in interval model updating [14]. Yin et al., 2019, designed an acceleration FR objective function and applied the Kriging model to replace this function [15]. Deng et al., 2017, used the radial basis function model to simplify the natural frequency calculation [16]. These simplified meta models are established by manual extracted FR features. It is unreliable to represent the complicated vibration characteristic and distinguish the inconspicuous vibration signal [17,18]. Therefore, how to remedy the data mining problem, reduce the need and effort in training samples collection, and improve the feature extraction reliability with limited amount of the training sample is becoming a particularly important topic in model updating.

Transfer learning (TL) has recently emerged as a beneficial approach to tackle the small samples problem [19]. It has attracted extensive attention in structure vibration analysis [20,21]. Transfer learning aims at using adequate source knowledge to solve a different but related target task. The target task can be completed by training a network with massive source domain data and fine-tune by deficient target domain data [22]. The TL algorithm has been widely studied in structure vibration analysis. Shen et al. trained a bearing fault diagnosis network by auxiliary data and similarity verify [23]. Zhang et al. trained a deep neural network with small target samples and abundant source samples in different working conditions [24]. Shao adopted ImageNet dataset [25] as the source data and time-frequency spectrum to classify mechanical faults [26]. 

In TL, domain adaptation (DA) is an effective strategy to decrease domain discrepancy. It is devoted to mapping sample features extracted from the source and target domain to a shared feature space [27]. In particular, maximum mean discrepancy (MMD)-based domain adaptation is widely used in TL [28]. Wen et al., 2019, proposed a DA method through weighted MMD term to lessen the discrepancy penalty [29] and tested with three datasets. Tang et al. [30] and Wang et al. [31] combined DA loss and classifier loss to design the network cost function. Yang et al., 2019, proposed a multi-layer domain adaptation method to reduce different domain discrepancy [32]. In those aforementioned TL research, the accessory source domain data are usually similar with target domain samples, such as the vibration signal obtained from the same experiment system under different working load [24] or the same machine signal in real-cases and laboratory [29]. However, in frequency-based model updating, sufficient similar structure frequency dataset is usually unavailable. The available vibration dataset like fault diagnose dataset are different from FR data in feature distribution. Hence, the domain adaptation method is critical to minimize the cross-domain distribution discrepancy and learn more cross-domain feature.

Therefore, to overcome the challenge of insufficient data problems encountered in model updating and improving the performance of cross-domain learning, a TL technique-based model updating method is proposed in this paper. The bearing fault diagnosis dataset from the Western Reserve University Bearing Data Center (CWRU) [33] is introduced as the source domain sample to help train the network. The main contributions of this method (termed as “TLNet method” in the following context) are as follows. (1) The deep transfer neural network is built with two branches. It has more capability in high-level feature extracting in source and target domain than manual feature methods. (2) Two-layer domain adaptation strategy based on maximum mean discrepancy is designed to adapt different datasets. (3) More precise inverse mapping is achieved under inadequate samples. Through this end-to-end framework, the model updating precision can be efficiently improved under sample-deficient problem compared to the method without transfer learning in reference [8].

This paper is organized as follows. Section 2 introduces the basic theory of model updating and transfer learning. Domain adaptation based on maximum mean discrepancy is also presented in Section 2. In Section 3, a detailed introduction of the proposed method and the network architecture manifests. A simulated satellite example is updated to demonstrate the feasibility of this method in Section 4. To evaluate the accuracy of the updated model, the model is validated in Section 5. Some conclusions are drawn for this research in Section 6.

## 2. Theoretical Background

### 2.1. Model Updating

In frequency response model updating methods, a residual function is usually designed to describe the deviation between simulate and experiment FR feature extracted manually. Then, the model updating problem will be transformed to a forward optimization problem where the optimal result is the final updating result. This optimization problem is as follows [7]:

(1) $$\theta =\mathrm{argmin}\;F,\;s.t.\;\theta_{iL}\leq \theta_{i}\leq \theta_{iU},$$

where **F** represents residual function and the optimization objective,$\;\theta_{iL}$ and $\theta_{iU}$ stand for the lower and upper bound of updating parameter $\theta_{i}$, respectively. The residual function is designed as follows:

(2) $$F=\overset{n_{\theta }}{\underset{n=1}{\sum }}\overset{n_{\omega }}{\underset{i=1}{\sum }}w_{i}{\left(\frac{A_{e}^{n}\left(\omega_{i}\right)-A_{a}^{n}\left(\omega_{i}\right)}{A_{e}^{n}\left(\omega_{i}\right)}\right)}^{2},$$

where $w_{i}$ denotes weights, $n_{\theta }$ is the number of measured sample location, $n_{\omega }$ is the number of selected frequency points of FR, $\omega_{i}$ is the selected frequency points, $A_{e}^{n}\left(\omega_{i}\right)$ and $A_{a}^{n}\left(\omega_{i}\right)$ denote the experimental and simulated acceleration frequency response amplitude at $\omega_{i}$.

Whereas, the residual function is inadequate to represent the structure dynamic feature and the manual feature extractor is inexact for complex FR feature. Hence, a high-precision inverse mapping from raw FR data to the updating parameter is proposed to overcome precision diminution in artificial feature extraction [8]. The inverse mapping can be formulated as follows:

(3) $$\theta =I_{A\theta }\left(A_{t}\right),$$

where $I_{A\theta }$ is the inverse mapping from experiment FR data $A_{t}$ to updating parameters θ. However, massive samples are essential to train a reliable network. Thus, transfer learning technique is adopted to reduce the quantity requirement of FR samples.

### 2.2. Transfer Learning

**Figure 1:**
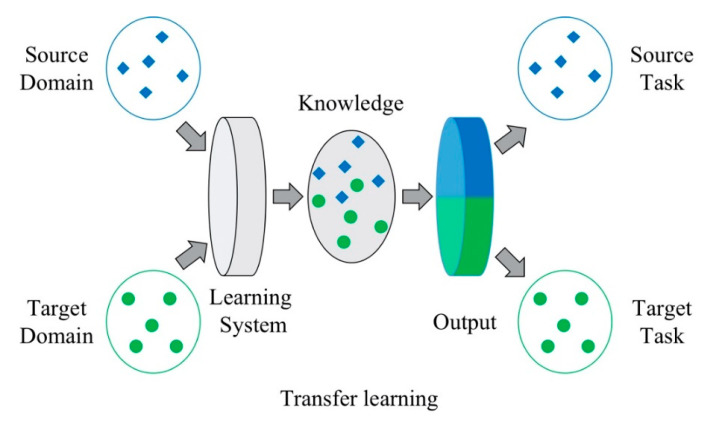
Illustration of transfer learning in model updating.

Transfer learning is proposed to apply the available knowledge and skills in previous domains to a novel domain. The known domain is source domain $D_{s}=\left\{\chi_{s},P_{s}\left(X_{s}\right)\right\}$ and the new domain with different distribution is the target domain $D_{t}=\left\{\chi_{t},P_{t}\left(X_{t}\right)\right\}$ [34], where $\chi_{s}$ and $\chi_{t}$ refer to the sample space of source and target domain. $X_{s}$ denotes the sufficient source domain sample for the source task $T_{s}$ and $X_{t}$ denotes the scanty target domain sample for the target task $T_{t}$, where $\chi_{s}\in X_{s}$ and $\chi_{t}\in X_{t}$. In this paper, the labeled bearing fault diagnosis data is in the sample space of source domain with the distribution $P_{s}\left(X_{s}\right)$. $P_{t}\left(X_{t}\right)$ stands for the distribution of the frequency response data in target sample space as described in Figure 1. In various research field, it is an effective strategy to fine-tune the pre-trained network when the source and target data has the same distribution [35]. However, in this paper, the feature distribution is different in FR data and fault vibration signal (namely $P_{s}\left(X_{s}\right)\neq P_{t}\left(X_{t}\right)$). Obviously, the model updating problem is inappropriate to be solved through directly fine-tuning a pre-trained network with target data. Therefore, the domain adaptation technology is necessary for feature mapping in different sample spaces.

### 2.3. Maximum Mean Discrepancy

Domain adaptation is an important technology in transfer learning [26]. It aims to map the data of source and target domain into a similar feature space and minimizing the discrepancy between the two feature spaces simultaneously. Then, the target knowledge in the shared feature space is learnt to improve the accuracy of the target task. Maximum mean discrepancy (MMD) is widely utilized in domain discrepancy quantification in DA, which is defined as follows [27]:

(4) $$Dis\left[\Psi_{s}\left(X_{s}\right),\Psi_{t}\left(X_{t}\right)\right]=\|\frac{1}{n_{s}}\overset{n_{s}}{\underset{r=1}{\sum }}\left(\Psi_{s}\left(\chi_{s_{r}}\right)\right)-\frac{1}{n_{t}}\overset{n_{t}}{\underset{r=1}{\sum }}\left(\Psi_{t}\left(\chi_{t_{r}}\right)\right){\|}_{H}^{2},$$

where $Dis\left[\Psi_{s}\left(X_{s}\right),\Psi_{t}\left(X_{t}\right)\right]$ is the discrepancy function, *H* represents the reproducing kernel Hilbert space (RKHS), $\Psi_{s}\left(X_{s}\right)$ and $\Psi_{t}\left(X_{t}\right)$ refer to the nonlinear mapping function in source and target domain from the original feature space to RKHS, $n_{s}$ and $n_{t}$ are the amount of source and target samples, $\chi_{s_{r}}$ and $\chi_{t_{r}}$ refer to the sample in $X_{s}$ and $X_{t}$, respectively. After feature mapping, the sample distribution in the new feature space will be diminished, namely $P_{s}\left(X_{s}\right)\approx P_{t}\left(X_{t}\right)$.

## 3. Procedure of Proposed Method

**Figure 2:**
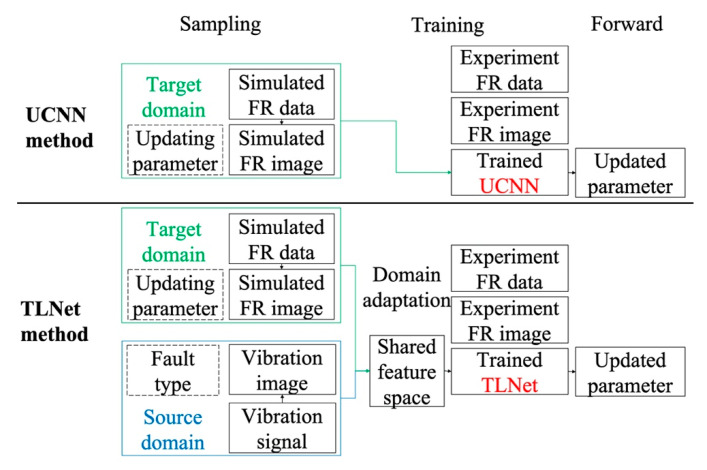
Flowchart of the UCNN [8] method and TLNet method.

In this section, the procedure of the TLNet method is introduced. In reference [8] (hereafter this text will be abbreviated as the UCNN method), a high precious updated model is achieved with sufficient training data. Therefore, the UCNN method is introduced as a comparison. The procedure of these two methods is displayed in Figure 2.

The detailed steps of the TLNet method are as follows:

Target domain data preparation

(1) Insufficient FR data are calculated from the finite element model. (2) The FR data are transformed to the FR image as network input and the corresponding updating parameters are labelled.

Source domain data preparation

(1) Massive fault diagnose vibration signal samples are acquired from the CWRU dataset. (2) Vibration samples are sliced to frames and the fault types are labelled.

Transfer learning

(1) A deep convolutional neural network with model updating and fault diagnose branch is built according to the target and domain sample dimension and label type. (2) The source and target features are extracted by the network. (3) Source and target features are embedded to a shared space through two domain adaptation. (4) The network is trained with the shared features.

Model updating

(1) The experiment FR data are transformed into the FR image as the network input. (2) The network forward propagation results are the final updated parameters.

It can be inferred that the two methods are similar in target data preparation, and forward propagation process, but different in transfer learning and network architecture. Source domain knowledge is learnt in auxiliary training in the TLNet method, which is the major difference between the two methods. Additionally, domain adaptation is necessary to learn the cross-domain assistant knowledge in the TLNet method.

### 3.1. Domain Adaptation

The features extracted from the network is used to quantify the discrepancy between source and target domain, as formulated in Equation (4). After embedding source and target domain data into a shared feature space, the distribution discrepancy is diminished by training the parameters of the nonlinear mapping function by minimizing MMD. Define matrices **K** and **L** as follows [34]:

(5) $$K=\left[\begin{array}{cc} K_{s,s} & K_{s,t}\\ K_{t,s} & K_{t,t} \end{array}\right],$$

and

(6) $$L=\left[\begin{array}{cc} \frac{1}{n_{s}^{2}} & -\frac{1}{n_{s}n_{t}}\\ -\frac{1}{n_{t}n_{s}} & \frac{1}{n_{t}^{2}} \end{array}\right],$$

where:

(7) $$\begin{array}{cc} K_{s,s}=\Psi_{s}\left(X_{s}\right)\Psi_{s}{\left(X_{s}\right)}^{T} & K_{s,t}=\Psi_{s}\left(X_{s}\right)\Psi_{t}{\left(X_{t}\right)}^{T}\\ K_{t,s}=\Psi_{t}\left(X_{t}\right)\Psi_{s}{\left(X_{s}\right)}^{T} & K_{t,t}=\Psi_{t}\left(X_{t}\right)\Psi_{t}{\left(X_{t}\right)}^{T} \end{array},$$

and:

(8) $$KL=\left[\begin{array}{cc} \frac{1}{n_{s}^{2}}\Psi_{s}\left(X_{s}\right)\Psi_{s}{\left(X_{s}\right)}^{T}-\frac{1}{n_{s}n_{t}}\Psi_{s}\left(X_{s}\right)\Psi_{t}{\left(X_{t}\right)}^{T} & \cdots \\ \cdots & \frac{1}{n_{t}^{2}}\Psi_{t}\left(X_{t}\right)\Psi_{t}{\left(X_{t}\right)}^{T}-\frac{1}{n_{t}n_{s}}\Psi_{t}\left(X_{t}\right)\Psi_{s}{\left(X_{s}\right)}^{T} \end{array}\right]$$

Then discrepancy function can be simplified as follows:

(9) $$Dis\left[\Psi_{s}\left(X_{s}\right),\Psi_{t}\left(X_{t}\right)\right]=tr\left(KL\right),$$

where tr(KL) stands for the trace of KL. Generally, $n_{s}$ and $n_{t}$ are the same in one batch. Therefore, the minimizing discrepancy function can then be eventually written as follows:

(10) $$Dis\left[\Psi_{s}\left(X_{s}\right),\Psi_{t}\left(X_{t}\right)\right]=tr\left(K\left[\begin{array}{cc} 1 & -1\\ -1 & 1 \end{array}\right]\right).$$

Considering the substantial distribution difference between original source and target data, the domain adaptation is utilized in two parts of the network to enhance the effect.

### 3.2. Source Domain Sample

The CWRU dataset is chosen as the auxiliary training knowledge. This source domain dataset was acquired from the accelerometers of the motor driving mechanical system [33]. Artificial damage on rolling bearing and single point fault was arranged. The damage diameters were 0.007 inch (0.1778 mm), 0.014 inch (0.3556 mm), and 0.021 inch (0.5334 mm). The damage points of the outer ring of the bearing at the drive end and the fan end are respectively placed at three different positions: 3 o’clock, 6 o’clock, and 12 o’clock [36]. In this paper, the bearing data will be classified to 10 categories: normal, inner ring, outer ring, ball with different damage positions and different damage diameters.

**Figure 3:**
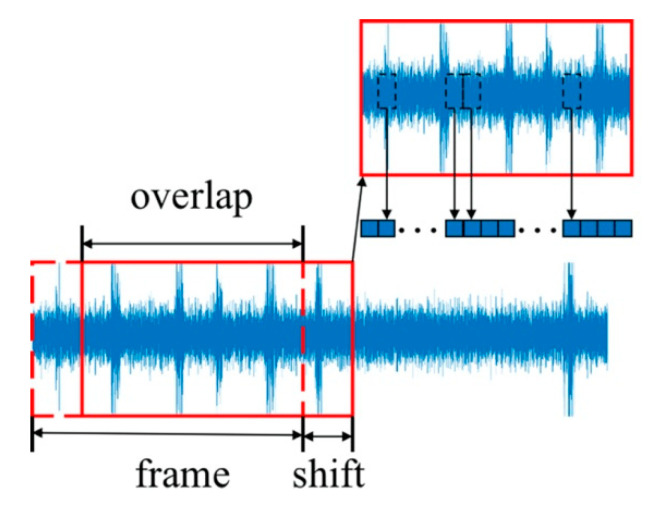
Frame of source domain data.

Since the dimensions of the source and target domain data are different, preprocessing is necessary before feature extraction. The source domain sample is continuous time-domain response, which needs to be Fourier transformed to the frequency-domain space. A part of the small stable signal with 1024 signal sampling points is sliced as a frame, as described in Figure 3. Frames are acquired overlapped, which is not continuously. The sample distance between the start positions of two adjacent frames is called the shift. The frame shift length in this paper is 512 sampling points, and the length of overlapping is 512 sampling points. It infers that the longer the overlap part is, the shorter the frame shift length will be, and more frames can be obtained. Therefore, using frames with overlap is an effectively sample expanding method. Finally, the source domain sample size is transformed to be 1 × 11 × 1024.

### 3.3. Target Domain Sample

**Figure 4:**
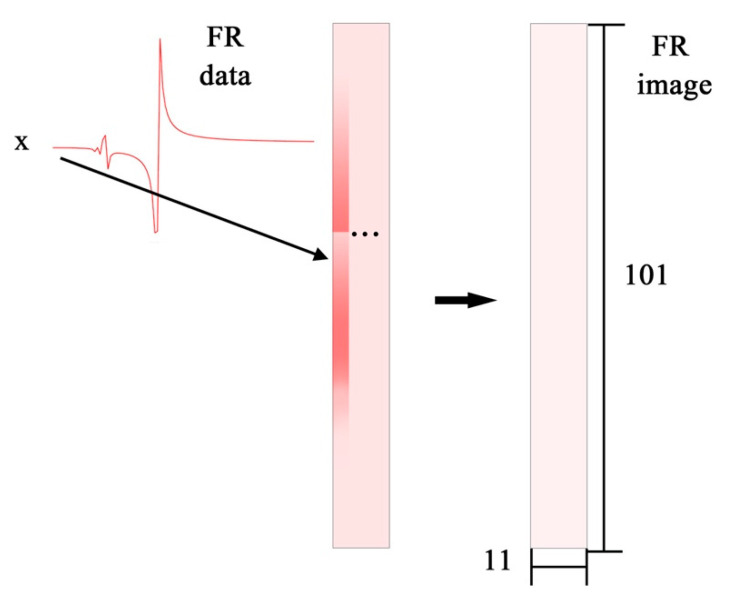
Illustration of FR data transforming to FR image.

In this paper, the FR image and corresponding updating parameters are the training pair for neural network training. In the TLNet method, a matrix transformed from the FR data will be firstly normalized into 0–255. After normalization, the FR data will be converted to multichannel image without artificial feature extraction, such as principal component analysis, reduction, and fitting. The channels, width, and height denote the acceleration orientation, number of the sampling location, and frequency measured sampling location of the FR signal, which is described in Figure 4. In Section 4, horizontal acceleration FR data of 11 sampling locations are measured from 0 to 100 Hz for every 1 Hz. Consequently, the FR size image is 1 × 11 × 101. The target training set is established by the simulation result of the updating model.

### 3.4. Network Architecture

**Figure 5:**
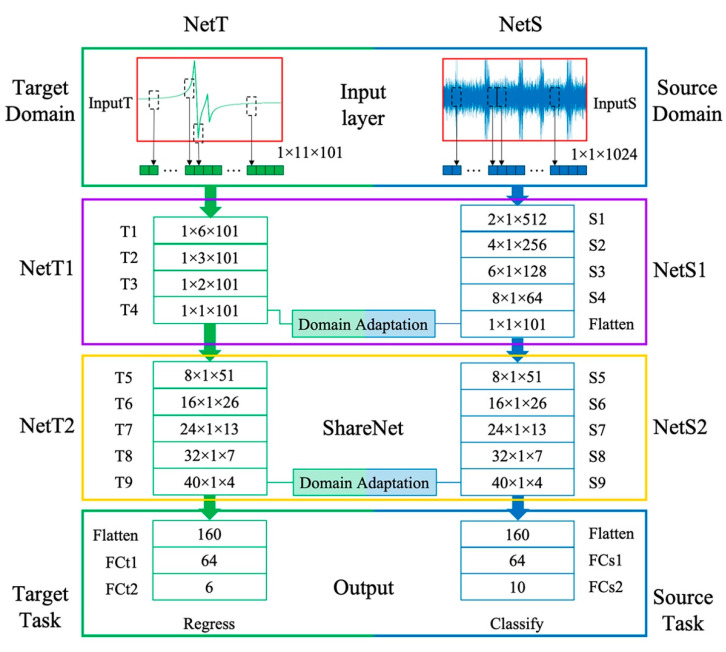
Network architecture of the TLNet.

In this paper, the deep convolutional neural network is adopted as the feature extractor for training samples. Aiming at taking advantage of target and source data, the network is designed to have two branches: target branch for model updating task and source branch for fault diagnose branch. The feature of FR data will be extracted layer-by-layer in the target branch of the network and the bearing vibration frames will be learnt in the source branch. Figure 5 displays the detail of the proposed network. The network structure is designed as follows:

1. NetT1 block: four convolutional layers block to extract feature of FR data at different sample locations and reshape the feature map to the size of 1 × 1 × 101.
2. NetS1 block: four convolutional layers with one flatten layer block to extract feature of bearing vibration and reshape the feature map to the same size as the output of NetT1 block.
3. NetT2 block: five layers network block to extract feature of NetT1 output.
4. NetS2 block: the same structure as NetT2 to extract feature of NetS1 output.
5. Output layers: a flatten layer followed by two fully connected layers to reshape the feature map to source and target task respectively.

In the TLNet method, the network is trained with scanty FR samples and massive fault diagnose samples. The training pairs in the two domains are sent to each branch of the network separately. In each branch, the low-level features are firstly extracted. Through convolution layers in the NetT1 and NetS1 block, the target features and the source features are transformed to the same size to compute domain discrepancy. Secondly, the features in the two domains are mapped to a shared feature space by first domain adaptation. Thirdly, the NetT1 and NetS1 output are sent to two network branches with the same structure (namely the ShareNet) to extract the deep-level feature. Fourthly, the second domain discrepancy is calculated, the output features of the two shared branches are mapped to a same feature space again by the second enhanced domain adaptation. Finally, the task of source and target domain is complete through output layers. The kernel size, stride size, and the channel number of TLNet parameter are listed in Table 1.

It can be inferred that the feature from the prior layer is sufficiently propagated to the post layer with the feature map in TLNet. The domain discrepancy is narrowed through two domain adaptation strategy. Eventually, the fault diagnosis task and the model updating task is completed through this network.

In this paper, the network is implemented onto the machine learning framework PyTorch 1.4.0 [37]. The first-order gradient-based stochastic optimization algorithm Adam is utilized in training [38].

### 3.5. Model Updating

After training, the measured FR image of the real structure will be sent to this trained model updating network. Then, the network is the value of target parameters, namely the final updated parameters of the observed FE model.

## 4. Case Study

**Figure 6:**
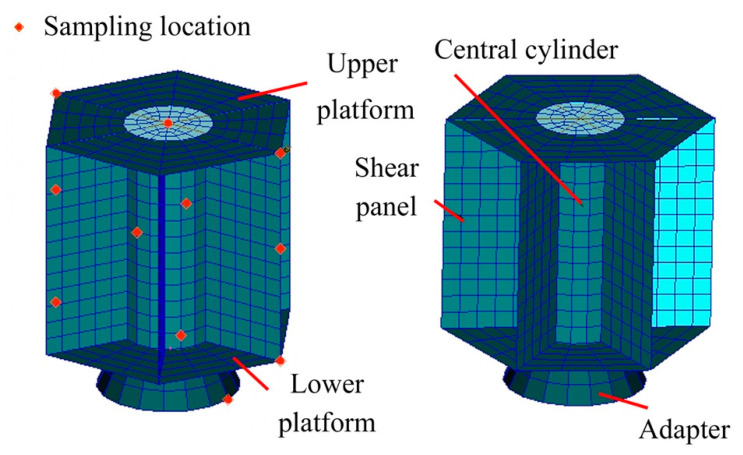
Finite element model of satellite and sampling location.

A satellite model is present to demonstrate the feasibility and effectiveness of the TLNet method. Figure 6 plots the FE model of the satellite and the sampling locations. 

### 4.1. Example Introduction

The updating parameters are the material parameter and the thickness of the structure: elastic model of the major structure $\theta_{1}$, density of the major structure $\theta_{2}$, thickness of the upper platform $\theta_{3}$, thickness of the lower platform $\theta_{4}$, thickness of the central cylinder $\theta_{5}$, and thickness of the shear panels $\theta_{6}$. The real value of these parameters is listed in Table 2. 

Horizontal acceleration FR data of 11 sampling locations are collected from 0 to 100 Hz for every 1 Hz by finite element software MSC. Patran and Nastran repeated simulations are implemented to build the data base of the initial model. FR data of X orientation is firstly normalized to 0–255 and then transformed into the FR image by sequence. In this section, FR data and the CWRU dataset are chosen to train the network. After training, the Z orientation data out of the training set are sent to the trained network in model validation. 

In this paper, the loss function (MMD2) is defined as follows:

(11) $$Loss=\eta_{1}Dis\left[\Psi_{s}\left(X_{s}\right),\Psi_{t}\left(X_{t}\right)\right]+\eta_{2}MSE\left(\theta_{t_{output}},\theta_{t_{label}}\right)+\eta_{3}CE\;+\eta_{4}Dis\left[{\tilde{\Psi }}_{s}\left({\tilde{X}}_{s}\right),{\tilde{\Psi }}_{t}\left({\tilde{X}}_{t}\right)\right],$$

where $\theta_{t_{output}}$ and $\theta_{t_{label}}$ represent network output and label in target domain, X and $\tilde{X}$ stand for the input of NetT1 and NetT2 (or NetS1 and NetS2) block, Ψ and $\tilde{\Psi }$ denote the output of NetT2 (or NetS2) block, CE refers to the cross-entropy loss for source classify task, MSE refers to the MSE loss for the target regress task, η stands for the feature loss factor of each loss function. In this paper, another loss function (MMDMSE) is designed as the comparison, which is defined as follows:

(12) $$Loss=\eta_{1}Dis\left[\Psi_{s}\left(X_{s}\right),\Psi_{t}\left(X_{t}\right)\right]+\eta_{2}MSE\left(\theta_{t_{output}},\theta_{t_{label}}\right)+\eta_{3}CE\;+\eta_{4}MSE\left[{\tilde{\Psi }}_{s}\left({\tilde{X}}_{s}\right),{\tilde{\Psi }}_{t}\left({\tilde{X}}_{t}\right)\right].$$

### 4.2. Result and Discussion

**Figure 7:**
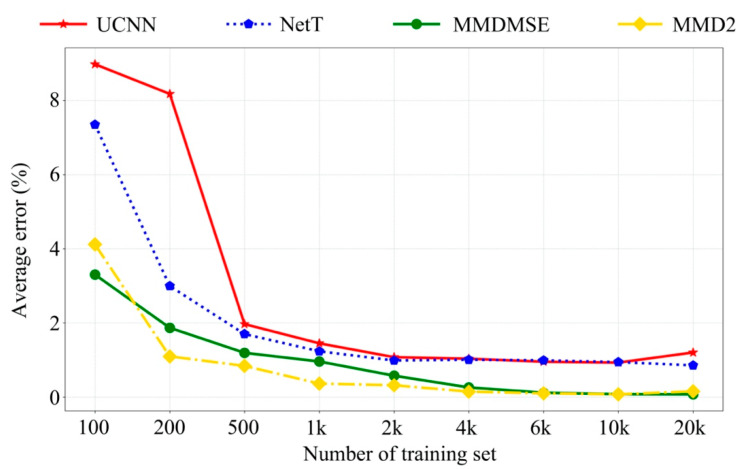
Average error with increasingly number of training set.

After training, the experiment FR data will be sent to the trained network, and the network output is the updating result. The parameters updated by two methods without TL (UCNN method and TLNet without source branch) and two methods with TL (MMDMSE and MMD2 loss function) are compared. Figure 7 shows the average errors corresponding to the mentioned four methods with the number of training sample increasing. From this figure, it is observed that the methods with domain adaptation (MMDMSE and MMD2) outperform with insufficient data. The average errors of TL methods are 3.302%, and 3.070% with 100 FR samples, while the average errors of the methods without transfer learning are 8.980% and 7.352%. With the increase of the sample amount, the average errors of those four methods all tend to decrease. When the sample number reaches to 4000 or even more, the accuracy appears to be stable. The result infers that the proposed method can performs better than the method without transfer learning when target training samples is extremely insufficient. This indicates that the representational ability of the proposed network can be improved with the help of vibration features learnt from the source data. Then, the precision of network output result can be improved even with inefficient data.

**Figure 8:**
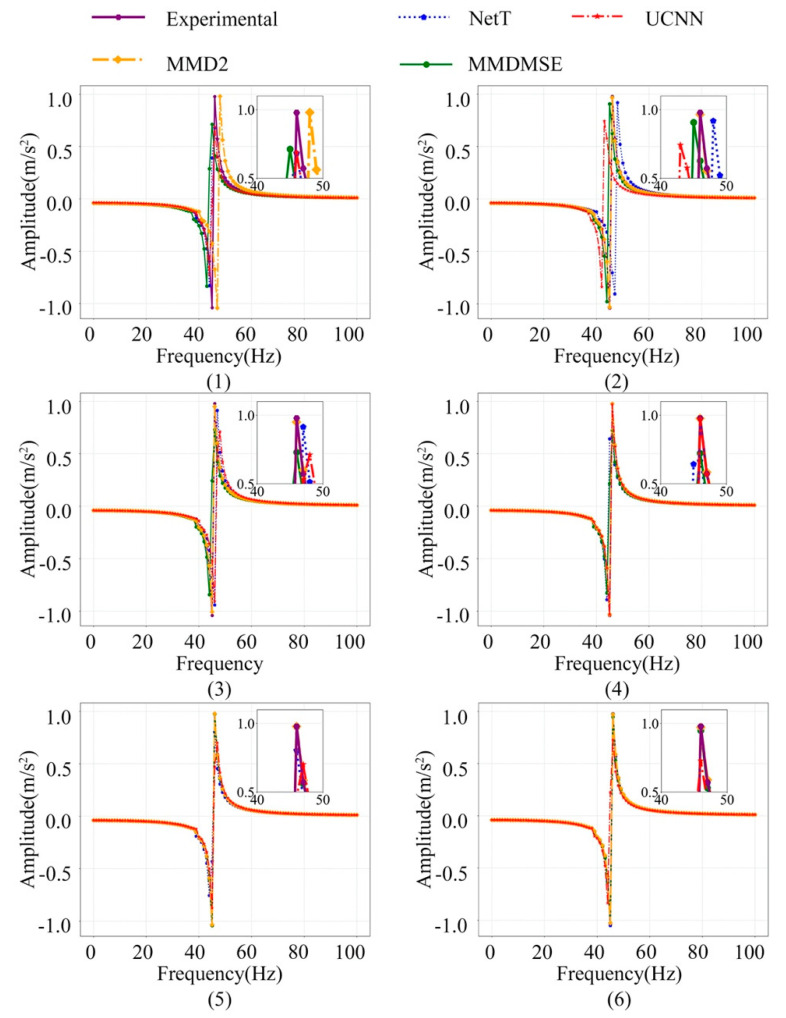
The FR signal of the No.1 sampling location at the X orientation sampling location with an increasing number of training samples ((1)–(6) represent for 100, 200, 500, 1000, 2000, 4000 samples).

The FR signal of the No.1 sampling location with increasing training sample size is plotted in Figure 8. Visually comparing the model frequency response with the experiment data implies that the updated model closely coincides with the real structure in the FR curve. The resonance peak amplitude and position resemble the experiment amplitude curve, which confirms that the model precision is substantially improved by the proposed method. With the increasing of the sample size, the FE model updated by TL methods still works better than those without TL technique. Specifically, the MMD2 loss is slightly better than the MMDMSE loss. The result suggests that the loss function based on MMD works well on mapping the domain-cross features to a more similar feature space, and it can also achieve better result in domain discrepancy diminution.

The final updating results with 4000 samples are presented in Table 2. The deviation between the updated and the real parameters is significantly lessened. The average error of the updated parameters with transfer learning is 0.257% and 0.145%, which is lower than the methods without transfer learning.

Furthermore, the frequency response assurance criterion (FRAC) is selected to assess the similarity between the updated simulation outputs and the experimental measurements [39]. When the data amount reaches to 200, the FRAC with MMD2 loss method is 0.963, while that with the UCNN method is 0.73. When the number of data volume increase to 2000, the FRAC is 0.999 with MMD2 and is 0.755 with the UCNN, respectively. This indicates that the outputs of the simulation model updated by the proposed method is closer to the experimental response, which proves the superiority of the TLNet method when the sample size is limited. A review of the updating results indicates that the model updating accuracy is successfully improved lacking samples.

## 5. Model Validation

**Figure 9:**
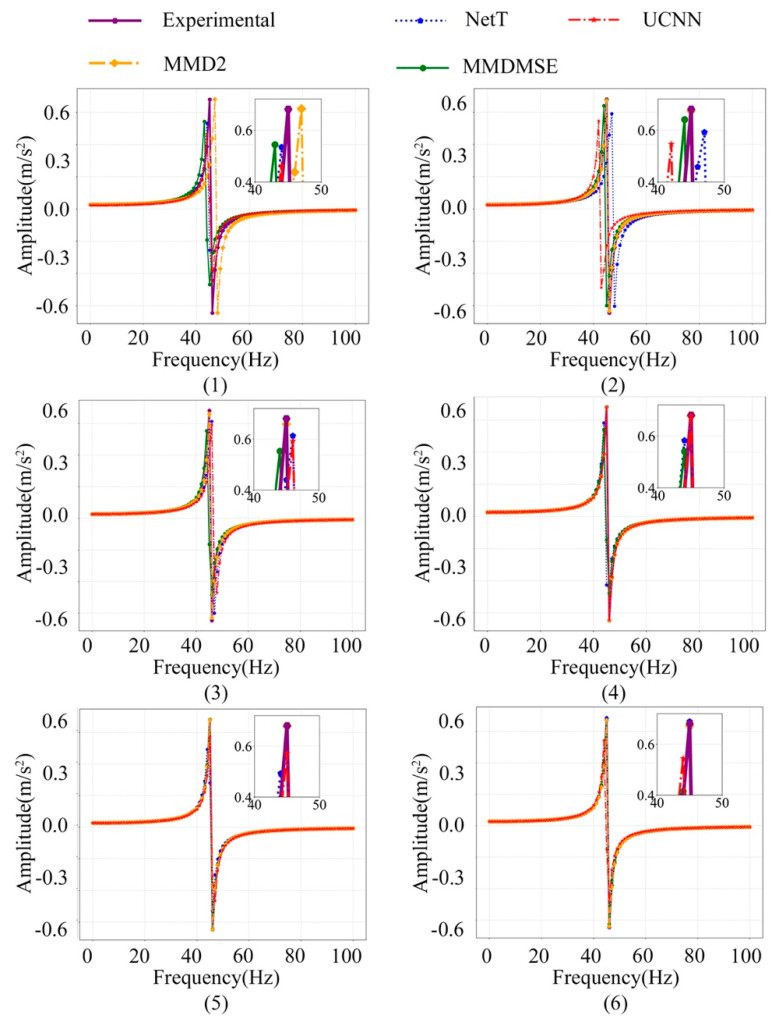
The FR signal of the No. 7 sampling location at the Z orientation sampling location with an increasing number of training samples ((1)–(6) represent for 100, 200, 500, 1000, 2000, 4000 samples).

To further evaluate the updated model, it is validated to by FR data at the Z orientation and the first five natural frequencies, which are unused in the training data. These two kinds of dynamic data are both excluded from the training set. Figure 9 displays the model frequency response at Z orientation corresponding to four updated FE models with above introduced methods, and meanwhile, the size of training sets increases gradually. For the situation of the 100 simple size, the simulated outputs, namely the FR signal of the FE model updated by MMD2 loss, can have a better effect in matching with the measured FR signal. It can be observed in this figure that the frequency response of the updated model coincides better with that of the real structure, though the Z orientation sample is excluded for training.

**Figure 10:**
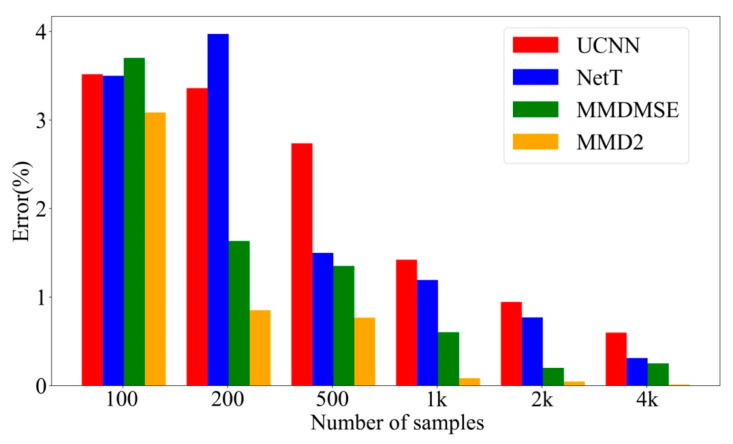
Average error of natural frequency.

Furthermore, the updated model is also validated by natural frequency. Figure 10 manifests the average error of the first natural frequency updated by 4 methods. It implies that the average error is lower in the MMD2 method with limited training samples. With training samples increasing, the model accuracy of MMD2 is still higher than the method without TL. Table 3 shows the natural frequency of the updated model with 4000 training samples. The average error of natural frequency of the MMD2 method is 0.012%, which is 0.586% lower than that of the UCNN method. This implies that the MMDMSE and MMD2 loss also have better performance in nature frequency prediction.

These validation results illustrate that TLNet method performs well in and out of the training set. The updated model can achieve better accuracy by using this method. The proposed approach has the capability to mitigate sample size requirement in model updating.

## 6. Conclusions

A model updating method based on transfer learning is proposed to tackle the small dataset problem. Using a two-branch deep neural network, a high-level feature extractor is employed to analyze the inverse relationship between FR data and the updating parameters. To make full use of the source domain knowledge, a two-layer domain adaptation strategy is adopted through mapping the cross-domain vibration feature into a shared space. Therefore, the cross-domain knowledge can be used in training a more reliable learning system. Finally, a high-precision inverse mapping from FR data to update parameters can be achieved.

The proposed method is tested by a satellite example with various number of training samples. Material or geometry parameters of the satellite model are updated. The results indicate that the proposed method has achieved higher-precision updating parameters. The model updated by the proposed method is more accurate than those updated by comparison methods without transfer learning. It can prove that the vibration feature from fault diagnose can be grasped in the learning system and it can provide more useful information for vibration analysis. Results also reveal that TLNet has higher ability in feature extraction than the networks trained only by target samples. Through this method, it can achieve significant superiority in model updating with insufficient data. The updated model can also have a more accurate prediction in response out of training set.

The future studies can be extended to applications that are sensitive to sample size, like the uncertainty model updating problem. It can be used to reduce sample need in uncertainty propagation analysis.

## Figures and Tables

**Table 1:** Details of the proposed network.

	NetT1					NetS1				
Layer name	InputT	T1	T1	T1	T1	InputS	S1	S2	S3	S4
Kernel size	-	(1,3)	(1,3)	(1,3)	(1,3)	-	(3,1)	(3,1)	(3,1)	(3,1)
Stride size	-	(1,2)	(1,2)	(1,2)	(1,2)	-	(2,1)	(2,1)	(2,1)	(2,1)
Channel	1	1	1	1	1	1	2	4	6	8
	**NetT2**					**NetS2**				
Layer name	T5		T6	T7	T8	T9		S5	S6	S7
Kernel size	(3,1)		(3,1)	(3,1)	(3,1)	(3,1)		(3,1)	(3,1)	(3,1)
Stride size	(2,1)		(2,1)	(2,1)	(2,1)	(2,1)		(2,1)	(2,1)	(2,1)
Channel	8		16	24	32	40		8	16	24

**Table 2:** Model updating result with 4000 samples.

Method	*θ*_1_ (10^10^ pa)	*θ*_2_ (10^3^ kg·m^−3^)	*θ*_3_ (mm)	*θ*_4_ (mm)	*θ*_5_ (mm)	*θ*_6_ (mm)	Average Error
Real	7.00	2.70	2.00	1.000	2.00	2.00	-
UCNN	6.947	2.677	1.979	0.994	2.959	1.969	1.032%
NetT	6.810	2.703	2.016	1.004	3.046	2.009	1.005%
MMDMSE	7.007	2.700	2.000	0.997	2.985	2.012	0.257%
MMD2	7.015	2.702	2.001	0.997	3.001	2.005	0.145%

**Table 3:** Model updating result with 4000 samples.

Natural Frequency	Experimental	UCNN	NetT	MMDMSE	**MMD2**
*f*_1_	19.11	19.22	19.16	19.16	19.12
*f_2_*	20.01	20.13	20.05	20.06	20.01
*f*_3_	23.31	23.43	23.35	23.36	23.31
*f*_4_	27.71	27.89	27.75	27.79	27.72
*f*_5_	28.07	28.26	28.31	28.15	28.07
Average error	-	0.598%	0.311%	0.250%	0.012%

## References

[1] Boulkaibet I., Mthembu L., Marwala T., Friswell M.I., Adhikari S. (2016). Finite element model updating using Hamiltonian Monte Carlo techniques. Inverse Probl. Sci. Eng..

[2] Modak S., Kundra T., Nakra B. (2000). Model updating using constrained optimization. Mech. Res. Commun..

[3] Yuan Z.X., Yu K.P. (2015). Finite element model updating of damped structures using vibration test data under base excitation. J. Sound Vib..

[4] Pradhan S.K., Modak S.V. (2018). A two-stage approach to updating of mass, stiffness and damping matrices. Int. J. Mech. Sci..

[5] Yang X., Guo X., Ouyang H., Li D. (2017). A new frequency matching technique for FRF-based model updating. J. Phys. Conf. Ser..

[6] Tran-Ngoc H., Khatir S., De Roeck G., Bui-Tien T., Nguyen-Ngoc L., Wahab M.A. (2018). Model Updating for Nam O Bridge Using Particle Swarm Optimization Algorithm and Genetic Algorithm. Sensors.

[7] Wang J., Wang C., Zhao J. (2017). Frequency response function-based model updating using Kriging model. Mech. Syst. Signal Process..

[8] Xinjie Z., Zhongmin D., Yanlin Z. (2019). A frequency response model updating method based on unidirectional convolutional neural network. Mech. Adv. Mater. Struc..

[9] Guo N., Yang Z., Jia Y., Wang L. (2016). Model updating using correlation analysis of strain frequency response function. Mech. Syst. Signal Process..

[10] Li W.-M., Hong J.-Z. (2012). Research on the iterative method for model updating based on the frequency response function. Acta Mech. Sin..

[11] Sipple J., Sanayei M. (2014). Finite element model updating of the UCF grid benchmark using measured frequency response functions. Mech. Syst. Signal Process..

[12] Ben Abdessalem A., El-Hami A. (2015). A probabilistic approach for optimising hydroformed structures using local surrogate models to control failures. Int. J. Mech. Sci..

[13] Wang F., Xu Y., Zhan S. (2016). Multi-scale model updating of a transmission tower structure using Kriging meta-method. Struct. Control. Heal. Monit..

[14] Fang S.E., Zhang G.-H., Lin Y.-Q., Zhang X.-H. (2015). Uncertain parameter identification using interval response surface model updating. J. Vib. Eng. Technol..

[15] Yin H., Ma J., Dong K., Peng Z., Cui P., Yang C. (2019). Model Updating Method Based on Kriging Model for Structural Dynamics. Shock. Vib..

[16] Deng Z., Guo Z., Zhang X. (2017). Interval model updating using perturbation method and Radial Basis Function neural networks. Mech. Syst. Signal Process..

[17] Kang J., Zhang X., Cao H., Qin S. (2020). Research on Multi-Alternatives Problem of Finite Element Model Updating Based on IAFSA and Kriging Model. Sensors.

[18] Machado T.H., Mendes R.U., Cavalca K.L. (2016). Directional frequency response applied to wear identification in hydrodynamic bearings. Mech. Res. Commun..

[19] Zhang L. (2019). Transfer Adaptation Learning: A Decade Survey. arXiv.

[20] Pan S.J., Yang Q. (2009). A Survey on Transfer Learning. IEEE Trans. Knowl. Data Eng..

[21] Tan B., Song Y., Zhong E., Yang Q. Transitive transfer learning. Proceedings of the 21st ACM SIGKDD International Conference on Knowledge Discovery and Data Mining.

[22] He M., Zhang J., Zhang S. (2019). ACTL: Adaptive Codebook Transfer Learning for Cross-Domain Recommendation. IEEE Access.

[23] Shen F., Chen C., Yan R., Gao R.X. Bearing fault diagnosis based on SVD feature extraction and transfer learning classification. Proceedings of the 2015 Prognostics and System Health Management Conference (PHM); Institute of Electrical and Electronics Engineers (IEEE).

[24] Zhang R., Tao H., Wu L., Guan Y. (2017). Transfer Learning With Neural Networks for Bearing Fault Diagnosis in Changing Working Conditions. IEEE Access.

[25] Deng J., Dong W., Socher R., Li L.J., Li K., Fei-Fei L. Imagenet: A large-scale hierarchical image database. In Proceeding of the 2009 IEEE Computer Society Conference on Computer Vision and Pattern Recognition (CVPR 2009).

[26] Shao S., McAleer S., Yan R., Baldi P. (2019). Highly Accurate Machine Fault Diagnosis Using Deep Transfer Learning. IEEE Trans. Ind. Inform..

[27] Long M., Cao Y., Wang J., Jordan M. (2015). Learning Transferable Features with Deep Adaptation Networks. arXiv.

[28] Ghifary M., Kleijn W.B., Zhang M. Domain Adaptive Neural Networks for Object Recognition. Proceedings of the 9th International Conference, EuroHepatic.

[29] Wen L., Gao L., Li X. (2019). A New Deep Transfer Learning Based on Sparse Auto-Encoder for Fault Diagnosis. Ieee Trans. Syst. ManCybern. Syst..

[30] Chen M., Tang H., Chen M. (2019). Transfer-learning based gas path analysis method for gas turbines. Appl. Eng..

[31] Wang Q., Michau G., Fink O. Domain adaptive transfer learning for fault diagnosis. In Proceeding of the 2019 Prognostics and System Health Management Conference (PHM-Paris).

[32] Yang B., Lei Y., Jia F., Xing S. (2019). An intelligent fault diagnosis approach based on transfer learning from laboratory bearings to locomotive bearings. Mech. Syst. Signal Process..

[33] Lou X., Loparo K.A. (2004). Bearing fault diagnosis based on wavelet transform and fuzzy inference. Mech. Syst. Signal Process..

[34] Pan S.J., Tsang I.W.-H., Kwok J.T., Yang Q. (2010). Domain Adaptation via Transfer Component Analysis. Ieee Trans. Neural Netw..

[35] Wang J., Chen Y., Yu H., Huang M., Yang Q. Easy Transfer Learning By Exploiting Intra-Domain Structures. In Proceeding of the 2019 IEEE International Conference on Multimedia and Expo (ICME).

[36] Jian X., Li W., Guo X., Wang R.-Z. (2019). Fault Diagnosis of Motor Bearings Based on a One-Dimensional Fusion Neural Network. Sensors.

[37] Gao X., Ramezanghorbani F., Isayev O., Smith J.S., Roitberg A.E. (2020). TorchANI: A Free and Open Source PyTorch-Based Deep Learning Implementation of the ANI Neural Network Potentials. J. Chem. Inf. Model..

[38] Kingma D.P., Ba J. (2014). A method for stochastic optimization. arXiv.

[39] Zang C., Gräfe H., Imregun M. (2001). Frequency–domain criteria for correlating and updating dynamic finite element models. Mech. Syst. Signal Process..

